# Seasonal variations in bathtub drowning deaths and the impact of outdoor temperatures: a nationwide time-series analysis with future projections

**DOI:** 10.1265/ehpm.25-00286

**Published:** 2025-12-04

**Authors:** Yoshiaki Tai, Kenji Obayashi, Yuki Yamagami, Keigo Saeki

**Affiliations:** Department of Epidemiology, Nara Medical University School of Medicine, Nara, Japan

**Keywords:** Bath-related deaths, Outdoor temperature, Drowning deaths, Attributable fraction

## Abstract

**Background:**

Globally, Japan has the highest drowning mortality among older adults, largely because of bathing customs. Although this mortality rate peaks in winter, the nationwide impact of outdoor temperature has not been quantified, and whether specific days carry greater risks for bathtub drowning deaths remains unclear. Therefore, we aimed to address these gaps using nationwide data from Japan.

**Methods:**

We collected daily data on outdoor temperatures and bathtub drowning deaths (from death certificates), along with population data, across 47 prefectures from 1995–2020. A time series regression model incorporating a cyclic spline for day-of-year and a cross-basis function for outdoor temperature was used to estimate seasonality and temperature attributable fractions (AFs). Prefecture-specific estimates were pooled using meta-analysis. National holidays were defined by the Act on National Holidays.

**Results:**

During the study period, 99,930 home bathtub drowning deaths were recorded. The AF for seasonality modelled with a cyclic spline for day-of-year was 77.8% (empirical confidence interval [eCI]: 76.7–78.8%), which decreased to 15.3% (eCI: 13.1–18.0%) after adjusting for outdoor temperature, indicating that outdoor temperature accounted for 80.3% of the seasonal effect. Elevated risks were observed on Sundays (relative risk = 1.16, 95% CI: 1.12–1.20), holidays (1.12, 95% CI: 1.08–1.16), New Year’s Day (1.72, 95% CI: 1.61–1.84), and New Year’s Eve (1.63, 95% CI: 1.52–1.74) in the adjusted model, which included a cyclic spline for day-of-year and a cross-basis function for outdoor temperature.

**Conclusion:**

Our findings highlight the importance of mitigating the impact of outdoor temperature on bath-related death risk. Identifying high-risk days can be used to help develop targeted preventive strategies.

**Supplementary information:**

The online version contains supplementary material available at https://doi.org/10.1265/ehpm.25-00286.

## Introduction

Hot-tub bathing at home is a deeply ingrained custom in Japanese culture [1], serving not only hygienic purposes but also as a means of bodily warming [2]. Typical bathing patterns in Japan involve set water temperatures of 40–42 °C with an average immersion time of 13 minutes and a frequency of 4.5 times per week in winter and 38–40 °C with a frequency of 3.0 times per week in summer, although the immersion duration in summer remains unreported [3]. A previous study reported that a lower indoor home temperature was associated with a greater degree of heat exposure during hot-tub bathing [4]. This practice, possibly through thermoregulatory mechanisms and subsequent physiological responses, has been linked to several favourable health outcomes, including lower nighttime blood pressure [5], better sleep [6, 7], and lower cardiovascular risk [8].

However, hot-tub bathing is also associated with a significant public health concern in Japan—bath-related deaths, which are defined as sudden deaths occurring before, during, or after bathing from any cause [9]. Japan reports the highest global drowning mortality among older adults [10], largely attributed to the widespread practice of hot-tub bathing. The estimated annual number of bath-related deaths in Japan reached approximately 17,000 in 2010, expected to increase to approximately 27,000 by 2035 because of population ageing [11]. Among individuals who experienced bath-related deaths, 79% showed signs of drowning, and 26% had blood ethanol levels above 0.5 mg/mL [12]. Notably, these deaths exhibit strong seasonal variation, with a reported 6.9-fold higher risk during winter than in summer [12]. Furthermore, an inverse association between outdoor temperature and bath-related deaths was observed in multiple prefectures [13–15].

Despite these observations, to our knowledge, no previous study has examined the time-series association between day-of-year and bath-related deaths across Japan or quantified the specific contributions of seasonality and ambient temperature to this mortality. The generation of such estimates could highlight the potential importance of mitigating the impact of outdoor temperature on the number of bath-related deaths. Furthermore, it remains unclear whether the number of bath-related deaths varies by specific days of the year, month, or week, potentially reflecting behavioural patterns related to bathing practices. Additionally, previous projections of bath-related deaths were based on limited prefectures and considered only demographic changes in Japan’s population [11], without incorporating the potential mitigating effects of global warming and rising ambient temperatures.

Therefore, the aims of this study were to (1) examine the seasonal patterns of bath-related deaths and estimate the attributable fractions (AFs) of seasonality and outdoor temperature; (2) identify monthly, weekly, and specific calendar days associated with elevated risk; and (3) project future bath-related deaths under various temperature scenarios, accounting for predicted changes in Japan’s population structure. We analysed exhaustive data on bathtub drowning deaths—the most common and reliably identifiable type of bath-related death recorded on death certificates—across Japan from 1995 to 2020.

## Methods

### Study design

For the population-level time series analyses, we collected daily data on bathtub drowning deaths, defined as cases classified under accidental drowning and submersion while in a bathtub (International Classification of Diseases, 10th Revision [ICD-10] code W65), and daily mean outdoor temperatures for all prefectures in Japan for the period from 1995 to 2020. To project future mortality, we additionally collected modelled temperature and population data for 2020 to 2069. The Ethics Committee of Nara Medical University approved the study protocol (approval no. 3557).

### Daily number of bathtub drowning deaths

We obtained death certificate data on W65-coded deaths that occurred in residential homes without restrictions on age or sex across Japan from January 1, 1995, to December 31, 2020, as provided by the Japanese Ministry of Health, Labour and Welfare under Article 33 of the Statistics Act. Data prior to 1995 were excluded because of the use of the ICD-9. We used the ICD-10 code W65, as it is the most specific and reliably identifiable code for bath-related deaths recorded on death certificates. However, it does not capture all bath-related deaths, such as those classified as cardiac arrest, stroke, or heat-related illness. We calculated the daily number of W65-coded deaths by prefecture, on the basis of the location reported on the death certificate, from 1995 to 2020.

### Daily mean outdoor temperatures by prefecture

The daily mean outdoor temperatures were obtained from the official website of the Japan Meteorological Agency [16]. The temperature data covered the period from December 21, 1994, to December 31, 2020, to account for lagged associations in the analysis. For each prefecture, the temperature of the most populous city was used as a representative value. Further geographic subdivision was not conducted because of the limited number of daily bathtub drowning deaths at the municipal level.

### Projected daily mean outdoor temperatures in Japan

We obtained modelled daily mean temperature data for Japan from January 1, 2020, to December 31, 2069, under the Shared Socioeconomic Pathway (SSP) scenarios from Phase 6 of the Coupled Model Intercomparison Project [17, 18]. We selected four emission scenarios—SSP1-2.6, 2-4.5, 3-7.0, and 5-8.5—which represent a range of future climate trajectories, from low (SSP1-2.6) to high (SSP5-8.5) greenhouse gas emissions. The temperature data were derived from a general circulation model simulation of MRI-ESM2-0 available through the Inter-Sectoral Impact Model Intercomparison Project [19, 20], with Japan selected as the country of interest. We truncated the temperature projections at 2069 because population forecasts are only available through 2070, and our results are summarized in 10-year intervals beginning in 2020; thus, 2060–2069 represents the final complete decade that can be analysed consistently.

### Population data by prefecture

Yearly population data for each prefecture in each year from 1995 to 2020 were obtained from the Japanese Population Census [21], which is conducted every five years with interpolated estimates for the intervening years. The dataset also included the proportion of individuals aged ≥65 years for each prefecture.

### Projected population in Japan

Projected population data for Japan from 2020 to 2069 were obtained from the National Institute of Population and Social Security Research website [22]. The dataset includes nine projection scenarios based on combinations of three birth rate and three mortality rate assumptions. For this study, we selected three scenarios representing distinct population trajectories: a high birth rate with low mortality (high population), medium birth and mortality rates (intermediate population), and a low birth rate with high mortality (low population). All the datasets also included the proportion of individuals aged ≥65 years in Japan.

### National holidays

Certain calendar days were designated national holidays, as stipulated by the Act on National Holidays (Act No. 178 of 1984, as subsequently amended) [23].

### Statistical analysis

Descriptive statistics are presented as the means (standard deviations [SDs]), medians (interquartile ranges [IQRs]) and counts (%). Missing values for daily mean temperature were imputed using the average daily mean temperature recorded on the same calendar date in previous years.

We assessed the seasonality of the daily number of bathtub drowning deaths by applying a time series regression with a cyclic spline for the day-of-year in each prefecture. We applied two time series regression models, with and without adjustment for daily outdoor temperature, as commonly used in previous studies [24, 25]. Model details are provided in the Supplemental Methods. Briefly, the model incorporated day-of-year as a cyclic spline function with four degrees of freedom, along with a cross-basis function of the daily mean outdoor temperature and its lagged effects, as specified in a distributed lag nonlinear model (DLNM) [26].

Prefecture-specific seasonality estimates were pooled using a multivariate meta-analysis of the fitted cyclic spline coefficients and their corresponding variance–covariance matrices to derive the average seasonal mortality pattern across Japan. From this pooled seasonal curve, we identified the peak and trough estimates of W65-coded mortality, along with their corresponding timing (day-of-year). The peak-to-trough ratio—the ratio of peak mortality to trough mortality—was then calculated, with 95% confidence intervals (CIs) derived from the summarized variance–covariance matrix of the spline coefficients.

Using the same seasonal curve, we also estimated the AF, defined as the proportion of deaths that could be avoided if the mortality risk remained at the level observed at the seasonal trough. The 95% empirical CIs (eCIs) for the peak and trough timings and the AF were obtained through Monte Carlo simulations [27–29]. In parallel, coefficients from prefecture-specific temperature–log-mortality associations, along with their variance–covariance matrices, were pooled using a multivariate meta-analysis. The resulting temperature–mortality association was then summarized as the cumulative exposure–response relationship, integrating the estimated effects over the defined lag period [30]. To assess temporal changes in the seasonality of bathtub-drowning deaths, we estimated period-specific AFs (1995–2003, 2004–2012, and 2013–2020) on the basis of a consistent time-series regression model in which the proportion of individuals aged ≥65 years was included to adjust for shifts in the age distribution and enable valid comparisons across periods.

Relative risks (RRs) for specific days, including national holidays and day-of-week, were estimated using generalized linear models with a quasi-Poisson distribution, incorporating the population of each prefecture in each year as an offset. Adjusted RRs were calculated by including the cyclic spline for seasonality (4 degrees of freedom for day-of-year) and the cross-basis function for daily mean outdoor temperature, both of which are detailed in the supplemental methods. When the adjusted RR for day-of-week was estimated, the model additionally included holidays and year as dummy variables. When the adjusted RRs for holidays were estimated, the model included strata for day-of-week, year, and their interaction to control for temporal confounding. Prefecture-specific estimates were pooled using a multivariate meta-analysis to derive overall summary estimates for Japan.

To project the future number of bathtub drowning deaths, we modified the previously described model to account for population ageing and future years. Model details are provided in the Supplemental Methods. Briefly, we extended the original model—used to assess the seasonality of bathtub drowning deaths—by including the proportion of individuals aged ≥65 years in Japan and the calendar year (ranging from 2020 to 2069) as additional covariates. Daily estimates were aggregated to calculate the total projected number of bathtub drowning deaths for each decade from the 2020s to the 2060s.

All the statistical analyses were conducted using R software version 4.4.3, with the ‘mixmeta’ package used for multivariate meta-analysis and the ‘dlnm’ package used for DLNMs [31, 32]. P < 0.05 (two-sided) was considered to indicate statistical significance.

## Results

We analysed a total of 446,359 prefecture-days, corresponding to the number of days between 1995 and 2020 multiplied by 47 prefectures. Missing values for daily mean temperature occurred on 101 days (0.023%) and were successfully imputed, as none fell in 1995 or on February 29, 1996—dates excluded from the imputation process. The daily mean outdoor temperatures ranged from −11.5 °C to 33.7 °C, with the 25th, 50th (median), and 75th percentiles at 8.2 °C, 16.2 °C, and 22.8 °C, respectively. During the study period, 99,930 bathtub drowning deaths at home were recorded. The age distribution of these deaths is shown in Fig. S1. Summary statistics for bathtub drowning deaths and daily mean temperatures by prefecture are presented in Table S1.

**Fig. 1:**
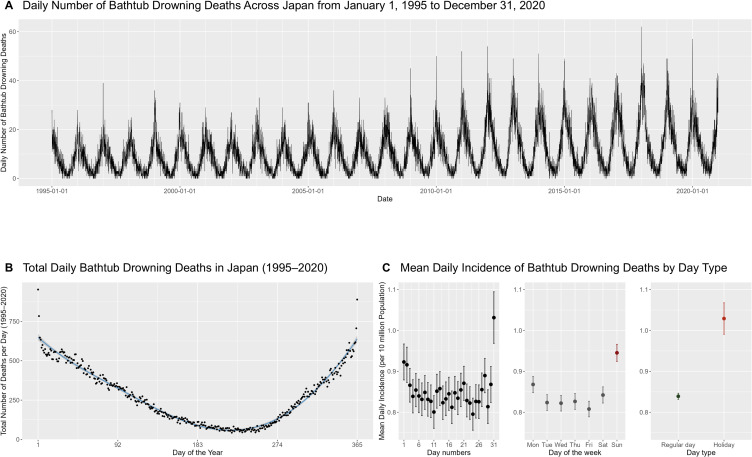
Daily Trends in Bathtub Drowning Mortality, 1995–2020 (A) Daily counts of bathtub drowning deaths in Japan from January 1, 1995, to December 31, 2020. (B) Total number of bathtub drowning deaths aggregated by day-of-year (1–365, removing February 29) across the study period. A cubic spline regression curve with knots placed at days 92, 183, and 274 is overlaid to illustrate seasonal variation. (C) Mean daily number of bathtub drowning deaths per 10 million people, with 95% confidence intervals (whiskers), stratified by day of the month, day of the week, and day type (regular weekdays vs. holidays).

Figure 1 presents the daily trends in bathtub drowning deaths. The number of deaths showed a recurring annual pattern, with a noticeable spike on the first day of each year (Fig. 1A). The total number of deaths by day-of-year followed an inverse U-shaped curve when modelled using a cubic spline, with outliers observed on January 1 and December 31 (Fig. 1B). The mean daily incidence per 10 million people was greater on the 31st day of each month than on the other days (Fig. 1C). However, this pattern disappeared after deaths that occurred between December 16 and January 15 were excluded (Fig. S2). By day-of-week, the mean daily incidence per 10 million people was highest on Sundays (0.95; 95% CI: 0.92–0.97) (Fig. 1C). Similarly, the incidence on holidays (1.03; 95% CI: 0.99–1.07) was higher than that on regular days (0.84; 95% CI: 0.83–0.85).

**Fig. 2:**
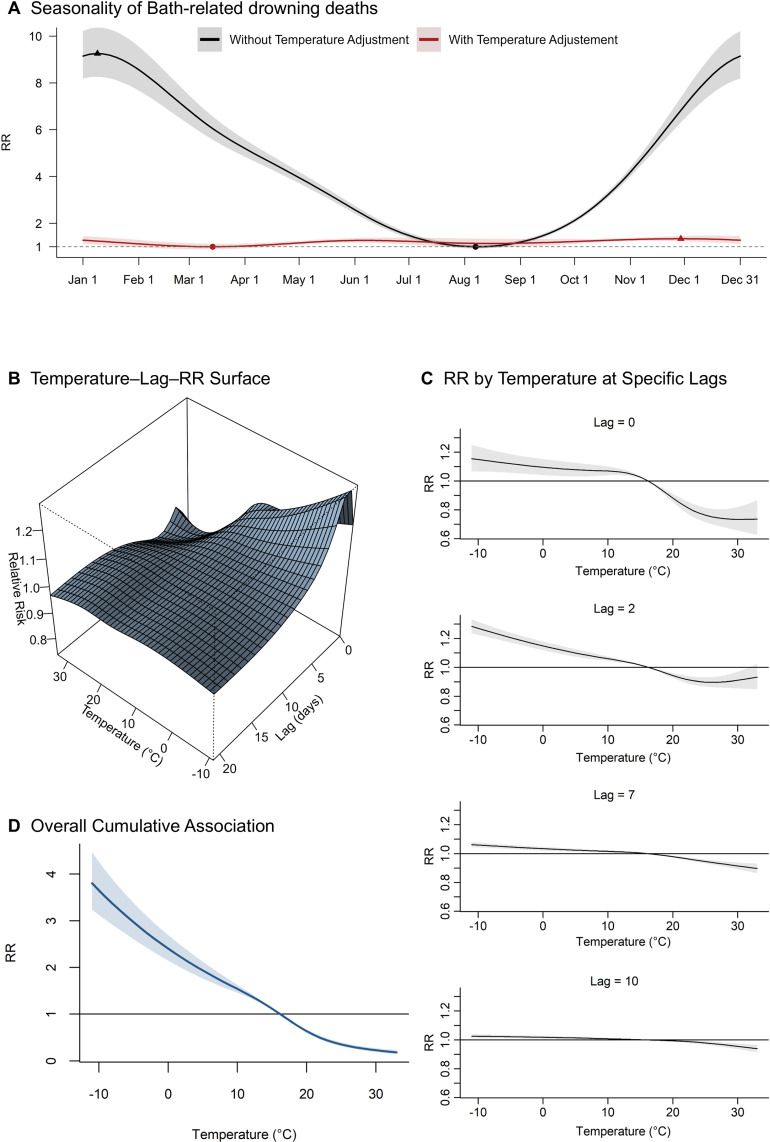
Seasonality and Outdoor Temperature-Related Risk of Bathtub Drowning Deaths (A) Seasonal trends in bathtub drowning deaths modelled using cyclic splines with 4 degrees of freedom for day-of-year, plotted with and without adjustment for daily mean outdoor temperature. The temperature-adjusted curve includes a cross-basis function accounting for lagged outdoor temperature effects up to 21 days. Triangles indicate the seasonal peaks, and dots indicate the troughs. (B) Bidimensional surface plot of the estimated RRs for bathtub drowning deaths by daily mean outdoor temperature and lag time, displayed in a 3D graph. (C) Lag-specific associations of outdoor temperature with the risk of bathtub drowning death at selected lag days (0, 2, 7, and 10). (D) Overall cumulative association between daily mean outdoor temperature and drowning risk, representing the sum of lag-specific effects over the 21-day lag period. Estimates from prefecture-specific analyses were pooled using multivariate meta-analysis. The shaded areas represent 95% confidence intervals. DLNM, distributed lag nonlinear model; RR, relative risk.

Figure 2A illustrates the seasonal variation in bathtub drowning deaths, with quantitative assessments summarized in Table 1. Before adjusting for outdoor temperature, a clear seasonal pattern was observed, with higher mortality in the cold season and lower mortality in the warm season. The peak and trough in mortality occurred on day 9 (eCI: 8–10) and day 219 (eCI: 220–221), respectively. After adjusting for outdoor temperature, the seasonality of mortality became less pronounced (Fig. 2A). Additionally, the estimated peak and trough timings (day 333, eCI: 164–348, and day 73, eCI: 67–226) cannot be strictly assigned to the winter and summer seasons, respectively, with increased uncertainty around these estimates. The peak-to-trough ratio substantially decreased after temperature adjustment, from 9.26 (95% CI: 8.42–10.19) to 1.34 (95% CI: 1.28–1.40). Similarly, the estimated AF was markedly reduced, with the difference in the AF of seasonality being 62.5 percentage points (77.8% to 15.3%), accounting for 80.3% of the seasonality-related AF observed without temperature adjustment. When stratified into 1995–2003, 2004–2012, and 2013–2020, the seasonal pattern of bathtub-drowning mortality remained stable, and period-specific AFs did not exhibit a clear monotonic trend (Fig. S3; Table S2).

**Table 1:** Assessment of Seasonality in Bathtub Drowning Deaths with and without Temperature Adjustment

**Temperature** **adjustment**	**Timings (day-of-year)** **(95% eCI)**		**Impact**	
	
	**Peak**	**Trough**	**Peak-to-trough ratio** **(95% CI)**	**Attributable fraction (%)** **(95% eCI)**
Unadjusted	9 (8, 10)	219 (220, 221)	9.26 (8.42, 10.19)	77.8 (76.7, 78.8)
Adjusted	333 (164, 348)	73 (67, 226)	1.34 (1.28, 1.40)	15.3 (13.1, 18.0)

CI, confidence interval; eCI, empirical confidence interval.

Figure 2B–D show the association between outdoor temperature and bathtub drowning mortality based on the DLNM. An inverse association between outdoor temperature and mortality was observed for temperatures below approximately 15 °C, particularly from lag 0 to lag 7 days, but this association largely disappeared by lag 10. Although the association appeared more prominent on lag 1 and lag 2, the 95% CIs overlapped with those of lag 0 (Fig. S4). Compared with the median outdoor temperature (16.2 °C), the pooled overall RRs were 2.40 (95% CI: 2.15–2.69) at 0 °C and 0.23 (95% CI: 0.19–0.27) at 30 °C (Fig. 2D).

The associations between day type and bathtub drowning mortality are presented in Table 2. After adjustment for seasonality, outdoor temperature, and strata, holidays were associated with a greater risk of bathtub drowning than were regular days, with an RR of 1.12 (95% CI: 1.08–1.16, P < 0.001). Similarly, New Year’s Day and New Year’s Eve presented significantly greater risks, with adjusted RRs of 1.72 (95% CI: 1.61–1.84) and 1.63 (95% CI: 1.52–1.74), respectively. In contrast, Coming-of-Age Day, a winter holiday, did not show a significantly higher risk after adjusting for the same variables. In the day-of-week analysis, the adjusted RRs for Monday, Saturday, and Sunday, compared with Friday, were 1.05 (95% CI: 1.02–1.08, P = 0.003), 1.04 (95% CI: 1.01–1.07, P = 0.015), and 1.16 (95% CI: 1.12–1.20, P < 0.001), respectively.

**Table 2:** Associations between Day Type and Bathtub Drowning Deaths

	**Unadjusted**			**Adjusted**		
	
	**RR**	**(95% CI)**	**P value**	**RR**	**(95% CI)**	**P value**
Day type						
Holiday (vs. Regular day)	1.23	(1.19–1.28)	<0.001	1.12	(1.08–1.16)	<0.001
New Year’s Day	3.59	(3.31–3.90)	<0.001	1.72	(1.61–1.84)	<0.001
New Year’s Eve	3.39	(3.15–3.65)	<0.001	1.63	(1.52–1.74)	<0.001
Coming-of-Age Day	2.13	(1.94–2.34)	<0.001	1.00	(0.91–1.09)	0.954

Day of the week						
Monday	1.06	(1.03–1.10)	<0.001	1.05	(1.02–1.08)	0.003
Tuesday	1.00	(0.98–1.03)	0.896	1.00	(0.98–1.03)	0.977
Wednesday	1.02	(0.99–1.05)	0.287	1.02	(0.99–1.05)	0.229
Thursday	1.01	(0.98–1.03)	0.710	1.01	(0.98–1.03)	0.666
Friday	Ref.	–	–	Ref.	–	–
Saturday	1.04	(1.01–1.07)	0.011	1.04	(1.01–1.07)	0.015
Sunday	1.16	(1.12–1.20)	<0.001	1.16	(1.12–1.20)	<0.001

RRs were estimated using a generalized linear model with quasi-Poisson regression. The adjusted model for day type included a cyclic spline for seasonality (4 degrees of freedom for day-of-year), a cross-basis function using a natural cubic spline for daily mean outdoor temperature, and strata defined by year, day of the week, and their interaction. The adjusted model for the day of the week included the same cyclic spline and temperature cross-basis, along with year and holiday (as factor variables). Prefecture-specific estimates were pooled using a multivariate meta-analysis.

**Fig. 3:**
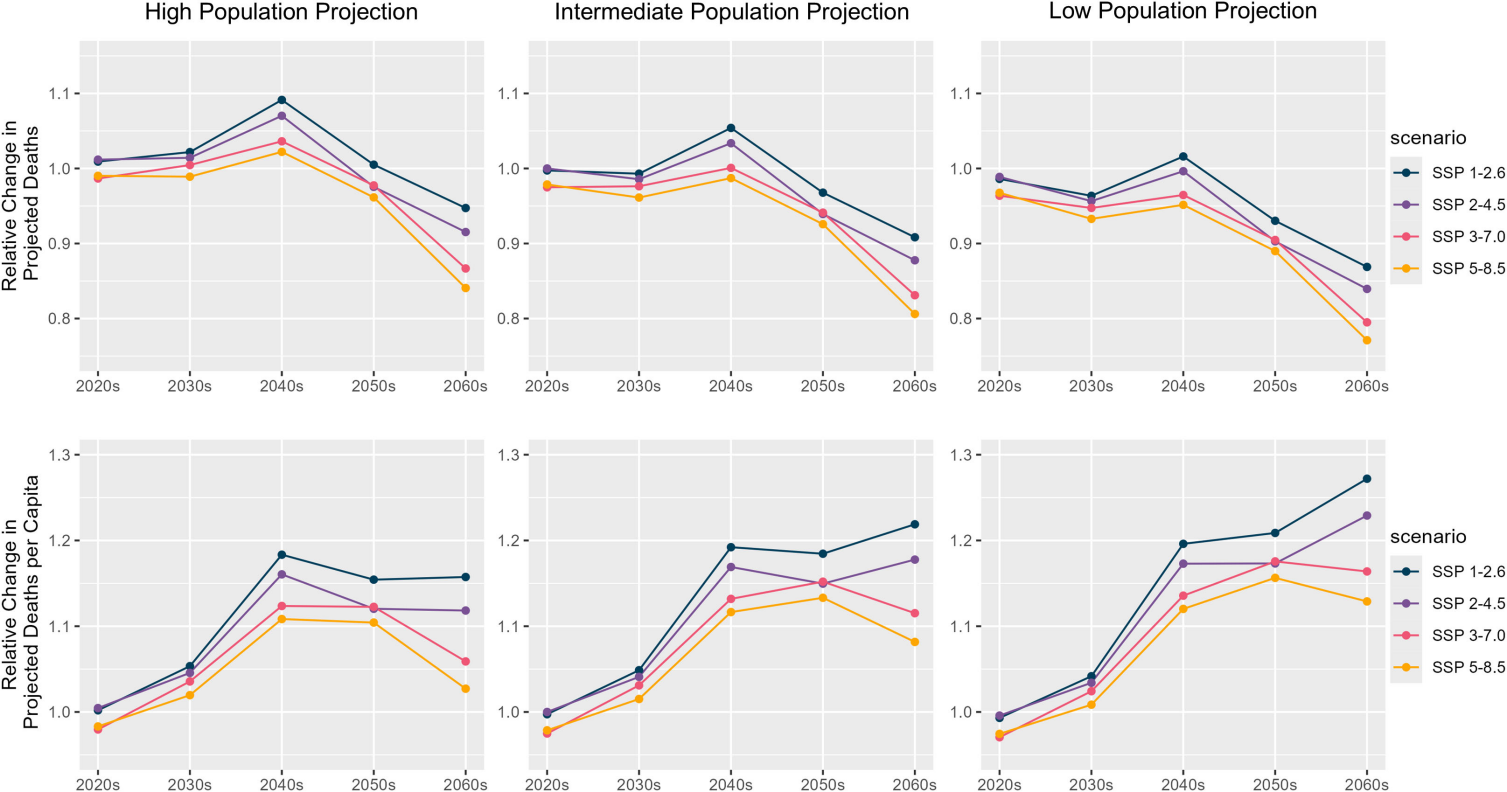
Projected Bathtub Drowning Deaths Under Future Population and Temperature Scenarios The projected number of bathtub drowning deaths was estimated as the sum of daily deaths derived from a model incorporating seasonality, a cross-basis function for daily mean temperature, and projections of the future Japanese population and the proportion of individuals aged ≥65 years. The population scenarios were classified as high, intermediate, or low on the basis of assumptions of birth and mortality rates: high birth rate and low mortality (high population), medium birth and mortality rates (intermediate population), and low birth rate with high mortality (low population).

The projected number of bathtub drowning deaths and the number of deaths per population under three population trajectories and four temperature scenarios, expressed as values relative to estimates for the 2020s under the intermediate population trajectory and the SSP2-4.5 temperature scenario, are shown in Fig. 3. The projected populations under the three scenarios used for mortality projections are detailed in Table S3. The total number of deaths begins to decline from the 2040s across all temperature scenarios, with the most pronounced decrease under the low population projection. In contrast, the projected mortality per population remains above 2020s levels through the 2060s, particularly in the low population scenarios under the SSP1-2.6 and SSP2-4.5 temperature pathways, where deaths per population continue to rise.

## Discussion

Using nationwide data on W65-coded deaths that occurred at home from 1995 to 2020, we identified clear seasonal patterns in bath-related drowning mortality, with an AF of 77.8%. The outdoor temperature accounted for 80.3% of this AF. The inverse association between outdoor temperature and bath-related drowning mortality was most pronounced from lag 0 to 2 days and gradually diminished towards lag day 10. We also found that New Year’s Day, New Year’s Eve, holidays, and Sundays were associated with significantly higher risks, even after adjusting for seasonality and outdoor temperature. Additionally, our projections across various temperature and population scenarios suggest that while the total number of deaths will decline—reflecting population decreases—per-capita deaths are expected to remain above the levels occurring in the 2020s through the 2060s under all temperature scenarios. To our knowledge, this is the first study to quantify the contributions of seasonality and outdoor temperature to bath-related deaths and to examine the lagged association between outdoor temperature and bath-related mortality.

Our findings underscore the importance of mitigating the impact of outdoor temperature on bath-related deaths, as reflected by the relatively high AF. In a previous study examining the seasonality of all-cause mortality using a similar statistical model, the peak-to-trough ratio and AF were estimated at 1.34 and 10.6%, respectively [25]. These figures contrast sharply with our estimates for bath-related deaths—9.26 for the peak-to-trough ratio and 77.8% for the AF—indicating a much stronger seasonal effect. Similarly, when comparing the temperature-related component of the AF from our study (62.5%) to previously reported AFs for cold- and heat-related mortality in Japan (9.81% and 0.32%, respectively) [33], both estimated using DLNMs, the impact of temperature on bath-related deaths appears disproportionately high. However, these estimates are not strictly comparable because the study periods, geographic coverage, and modelling strategies differ.

From a practical perspective, although the outdoor temperature cannot be directly controlled, its influence on bath-related drowning may be reduced by managing the indoor temperature and minimizing individual cold exposure. Elevated bathtub water temperatures during colder seasons may lead to excessive heat loads [4, 34], potentially triggering a cascade of physiological responses—hyperthermia, cardiovascular instability, consciousness disturbance, and subsequent drowning [11, 35]. A previous study revealed that the inverse association between outdoor temperature and bath-related heat exposure (measured by the maximum body surface temperature during bathing) was attenuated at relatively high indoor temperatures [4]. Similarly, a higher prebathing body surface temperature weakened the inverse association between the indoor temperature and bath-related heat exposure. These findings, along with our findings, underscore the importance of managing cold exposure at both the environmental and individual levels to reduce the risk of bath-related mortality.

The identified days with elevated RRs of bathtub drowning deaths may be linked to previously reported risk factors and offer practical value for prevention. A prior study revealed an inverse association between bath-related mortality and the availability of nursing care services that provide bathing support [36], suggesting that reduced service provision on Sundays and holidays—including the year-end and New Year periods—may contribute to the increased risk on these days. Additionally, the custom of daytime alcohol consumption on New Year’s Day in Japan may increase the risk of bath-related drowning [37], as previous studies have reported elevated blood ethanol levels among decedents [12, 14]. Furthermore, delays in seeking treatment during holiday periods may increase the risk of bathtub drowning [38]. In contrast, the non-significant result for Coming-of-Age Day suggests that holiday status does not uniformly increase risk. The pooled holiday effect may be driven by New Year’s Day (and New Year’s Eve), whereas Coming-of-Age Day, which focuses on younger celebrants, likely minimally influences daytime alcohol consumption and bathing behaviours among older adults. Although the underlying mechanisms could not be established in our study, the elevated risk on these days—independent of seasonality and temperature—should be acknowledged, and greater awareness should be raised about their potential danger.

Our projections of future bathtub drowning deaths highlight the continued importance of addressing this issue. Even under the highest mortality and greenhouse gas emission scenarios, the number of deaths per population is not expected to fall below 2020s levels until the 2060s. This suggests that the burden on healthcare systems—including forensic pathologists, medical examiners, and first responders—will remain unchanged relative to population size [12, 35, 39]. This is particularly concerning in suburban areas of Japan without medical examiner systems, where general physicians are often responsible for determining causes of death without specialized training [35]. In such settings, where expanding these systems remains challenging, prioritizing preventive measures for bath-related deaths is essential.

Although we focused on W65-coded deaths—the most common manner-of-death code for bath-related deaths—we acknowledge that this classification may include cases with other underlying causes, such as sudden cardiac arrest, myocardial infarction, arrhythmia, or stroke occurring during bathing. This limitation is particularly relevant when autopsies or postmortem imaging are not performed [40], as diagnosing drowning remains difficult even with autopsy due to the low prevalence of definitive signs [41]. Classification uncertainty is especially pronounced among older decedents, who are less likely to undergo autopsy [12]. Consequently, the estimated peak-to-trough ratio (9.26, [95% CI: 8.42–10.19]) might differ if all bath-related deaths were included. However, the inclusion of cardiac and cerebrovascular deaths would likely dilute—rather than amplify—the pronounced peak-to-trough ratio observed for bathtub drowning deaths, as these causes typically exhibit only modest winter excesses (22% to 78% increase) [42].

A key strength of our study is the use of exhaustive data from all Japanese prefectures (1995–2020) and the combined application of a cyclic spline for seasonality and a DLNM for outdoor temperature, enabling generalizable results and an unbiased decomposition of each factor’s AF, thereby providing robust evidence for public health planning.

However, our study has several limitations. First, the analysis was conducted at the population level and did not account for individual-level exposures, such as ambient or body surface temperature, or thermal conditions during bathing, although these factors are difficult to measure in large-scale, real-world settings. Second, the estimated AF for the outdoor temperature may partially reflect the effect of the indoor temperature, which was not included in the models but was moderately correlated with the outdoor temperature in certain ranges [43]. Third, the reliance on W65-coded deaths may have introduced classification bias, as previously discussed. Fourth, outdoor temperature data were linked to mortality by date of death rather than the date of incident, potentially leading to exposure misclassification. However, the use of a 21-day lag structure in the DLNM likely mitigated this bias in the estimation of peak-to-trough ratios and AFs. Moreover, this mismatch is expected to be minimal, as 99.6% of bath-related deaths occur within 24 hours of bathing [14].

In conclusion, we identified pronounced seasonal patterns in bath-related drowning mortality and a substantial contribution of outdoor temperature to this risk. We also found that certain calendar days are associated with elevated mortality, independent of seasonality and temperature effects. Additionally, our projections suggest that the number of deaths per population will likely persist through the 2060s, even under high greenhouse gas emission scenarios. These findings may inform future cluster-level intervention studies and guide public health strategies to address the ongoing burden of bath-related deaths.

## Abbreviations

AF: attributable fraction
DLNM: distributed lag nonlinear model
ICD: International Classification of Diseases
RR: relative risk
SSP: shared socioeconomic pathway

## Supplementary information

**Additional file 1:** Supplemental methods. **Fig. S1.** Age group distribution of bathtub drowning deaths that occurred at home (1995–2020). **Fig. S2.** Mean daily incidence of bathtub drowning deaths by day of the month, excluding December 16 to January 15. **Fig. S3.** Seasonal trends in bathtub drowning deaths by period. **Fig. S4.** Relative risk of bathtub drowning deaths by outdoor temperature: comparison of lag 0 vs. lag 1 and lag 0 vs. lag 2. **Table S1.** Summary statistics of bathtub drowning mortality and daily mean outdoor temperature by prefecture (1995–2020). **Table S2.** Seasonality of bathtub drowning deaths with and without temperature adjustment by period. **Table S3.** Projected population and proportion of individuals aged ≥65 years across three scenarios.

## Declarations

### Availability of data and material

The individual-level vital statistics data used in this study cannot be shared, as the authors do not possess the rights to distribute them. However, the deidentified public data and accompanying data dictionary employed in the analysis are publicly available and can be accessed directly without the need for a formal request.
