# Supplementary material for: Seasonal variations in bathtub drowning deaths and the impact of outdoor temperatures: a nationwide time-series analysis with future projections
Source: Environ Health Prev Med. 2025 Dec 4;30:99. doi: 10.1265/ehpm.25-00286 (PMC12698363; doi:10.1265/ehpm.25-00286)
Supplement: Supplementary file 1 — Additional file 1: Supplemental methods. Fig. S1. Age group distribution of bathtub drowning deaths that occurred at home (1995–2020). Fig. S2. Mean daily incidence of bathtub drowning deaths by day of the month, excluding December 16 to January 15. Fig. S3. Seasonal trends in bathtub drowning deaths by period. Fig. S4. Relative risk of bathtub drowning deaths by outdoor temperature: comparison of lag 0 vs. lag 1 and lag 0 vs. lag 2. Table S1. Summary statistics of bathtub drowning mortality and daily mean outdoor temperature by prefecture (1995–2020). Table S2. Seasonality of bathtub drowning deaths with and without temperature adjustment by period. Table S3. Projected population and proportion of individuals aged ≥65 years across three scenarios. [file ehpm-30-099-s001.docx]

**Supplemental materials**

**Supplemental methods**

***Statistical analysis***

We applied two time series regression models, with and without adjustment for daily outdoor temperature, as commonly used in previous studies:^1,2^

$$log(E\left( Y_{t} \right))= \alpha+cs\left( {Doy}_{t} \right)+ \lambda\cdot{Strata}_{t}+offset(log\left( {Pop}_{t} \right))$$

$$log(E\left( Y_{t} \right))= \alpha+cs\left( {Doy}_{t} \right)+ \lambda\cdot{Strata}_{t}+cb\left( {Temp}_{t,l} \right)+offset(log\left( {Pop}_{t} \right))$$

In these models, $Y_{t}$ denotes the number of bath-related deaths on day $t$, assumed to follow a quasi-Poisson distribution. The term $\alpha$ represents the intercept. $cs\left( {Doy}_{t} \right)$ is a cyclic spline function with four degrees of freedom for the day-of-year (ranging from 1 to 366), with days after March 1 shifted by one day in nonleap years to align the seasonal patterns across years. ${Strata}_{t}$ is a categorical variable accounting for year, day-of-week, and their interaction to control for long-term trends and weekly variations. When included, $cb\left( {Temp}_{t,l} \right)$ represents a cross-basis function of the daily mean outdoor temperature on day $t$ and its lagged effects over $l$ days, as implemented in distributed lag nonlinear models (DLNMs).^3^ For the exposure‒response relationship, we used a natural cubic B-spline with three internal knots placed at the 25^th^, 50^th^, and 75^th^ percentiles of the outdoor temperature distribution. The lag‒response relationship was modelled using a cubic B-spline with three degrees of freedom, extending up to 21 lags. The population size for each prefecture was included as an offset term using $log\left( {Pop}_{t} \right)$, assuming that the population remains constant within each calendar year because only annual population data were available.

The model used to project future bathtub drowning deaths was specified as follows:

$$\log\left( E\left( Y_{t} \right) \right)=\left. \alpha+ cs\left( {Doy}_{t} \right)+ \gamma\cdot{Dow}_{t}+ \right. \beta_{year}\cdot{Year}_{t}+cb\left( {Temp}_{t,l} \right)+\beta_{P65}{P65}_{t}+ offset(log\left( {Pop}_{t} \right))$$

In this model, ${Dow}_{t}$​ denotes the day-of-week as a set of dummy-coded variables, and ${Year}_{t}$ represents the calendar year on day $t$, treated as a continuous variable to account for years beyond 2020. ${P65}_{t}$ is the proportion of individuals aged ≥65 years and is used to capture the effect of population ageing. We applied this model separately to each prefecture and then pooled the results using a multivariate meta-analysis to obtain overall summary coefficients for Japan. We subsequently substituted the projected values for the daily outdoor temperature, total population, proportion of individuals aged ≥65 years in Japan, and calendar year (from 2020 to 2069) into the fitted model to estimate the predicted daily number of bathtub drowning deaths. By aggregating these daily estimates, we calculated the total number of projected deaths for each decade from the 2020s to the 2060s.

References

1. Madaniyazi L, Armstrong B, Chung Y, et al. Seasonal variation in mortality and the role of temperature: a multi-country multi-city study. *International journal of epidemiology.* 2022;51(1):122-133.

2. Madaniyazi L, Tobias A, Kim Y, Chung Y, Armstrong B, Hashizume M. Assessing seasonality and the role of its potential drivers in environmental epidemiology: a tutorial. *International journal of epidemiology.* 2022;51(5):1677-1686.

3. Gasparrini A, Armstrong B, Kenward MG. Distributed lag non-linear models. Stat Med. 2010;29(21):2224-2234.

**Fig. S1**. Age group distribution of bathtub drowning deaths that occurred at home (1995–2020)


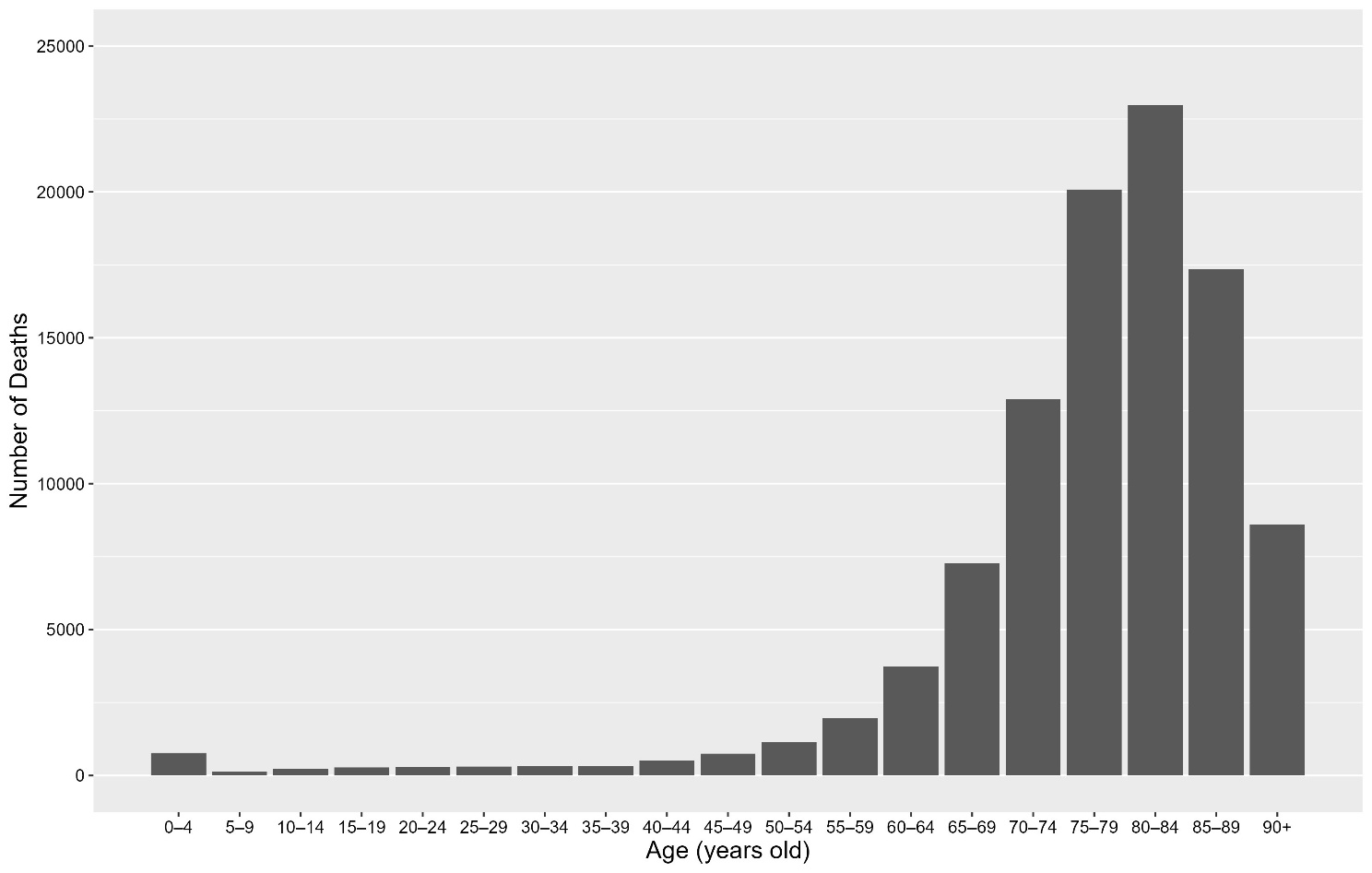


**Fig. S2**. Mean daily incidence of bathtub drowning deaths by day of the month, excluding December 16 to January 15


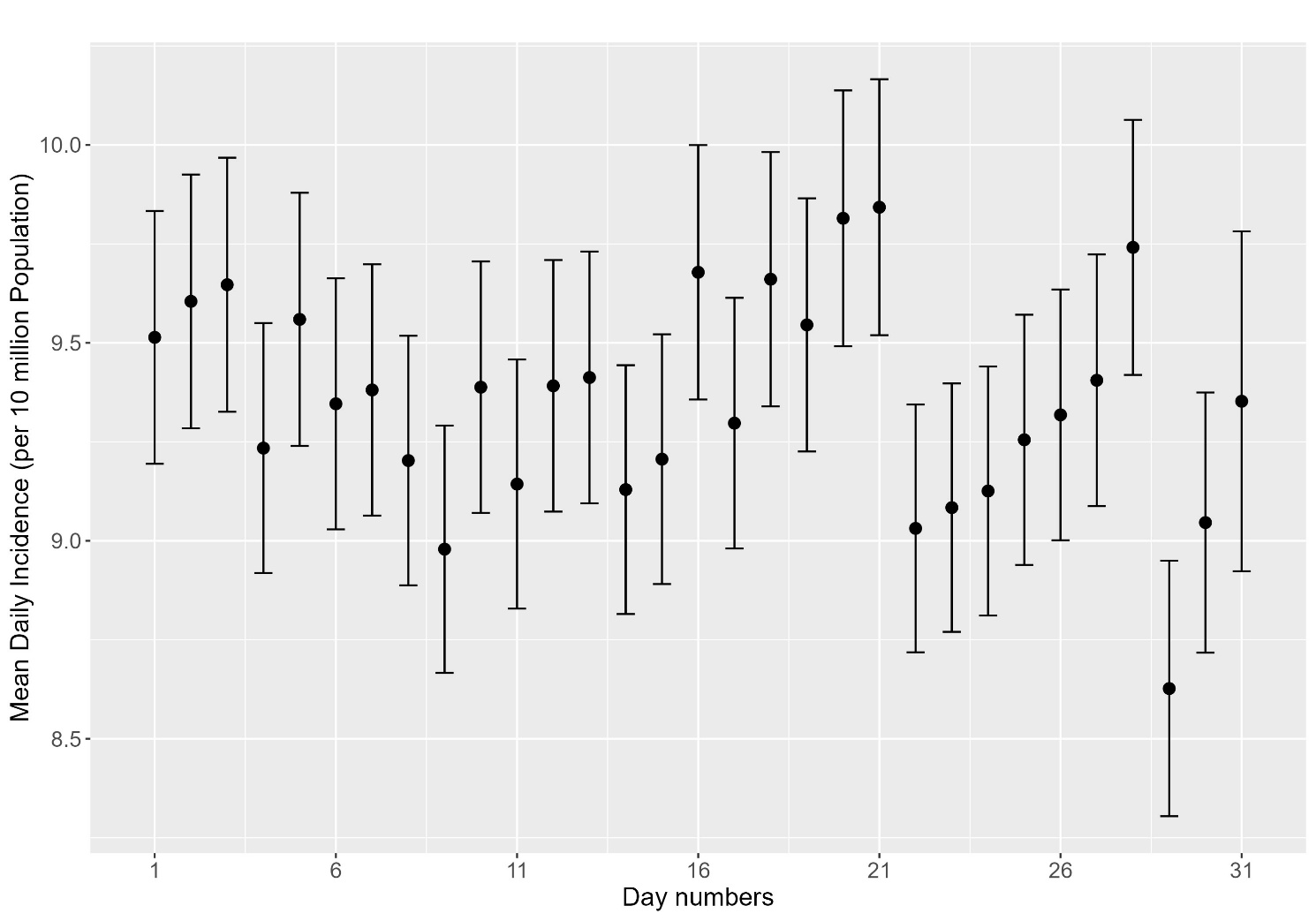


Mean daily number of bathtub drowning deaths per 10 million population, with 95% confidence intervals (whiskers), stratified by day of the month, excluding the period from December 16 to January 15 across the entire study period.

**Fig. S3**. Seasonal trends in bathtub drowning deaths by period


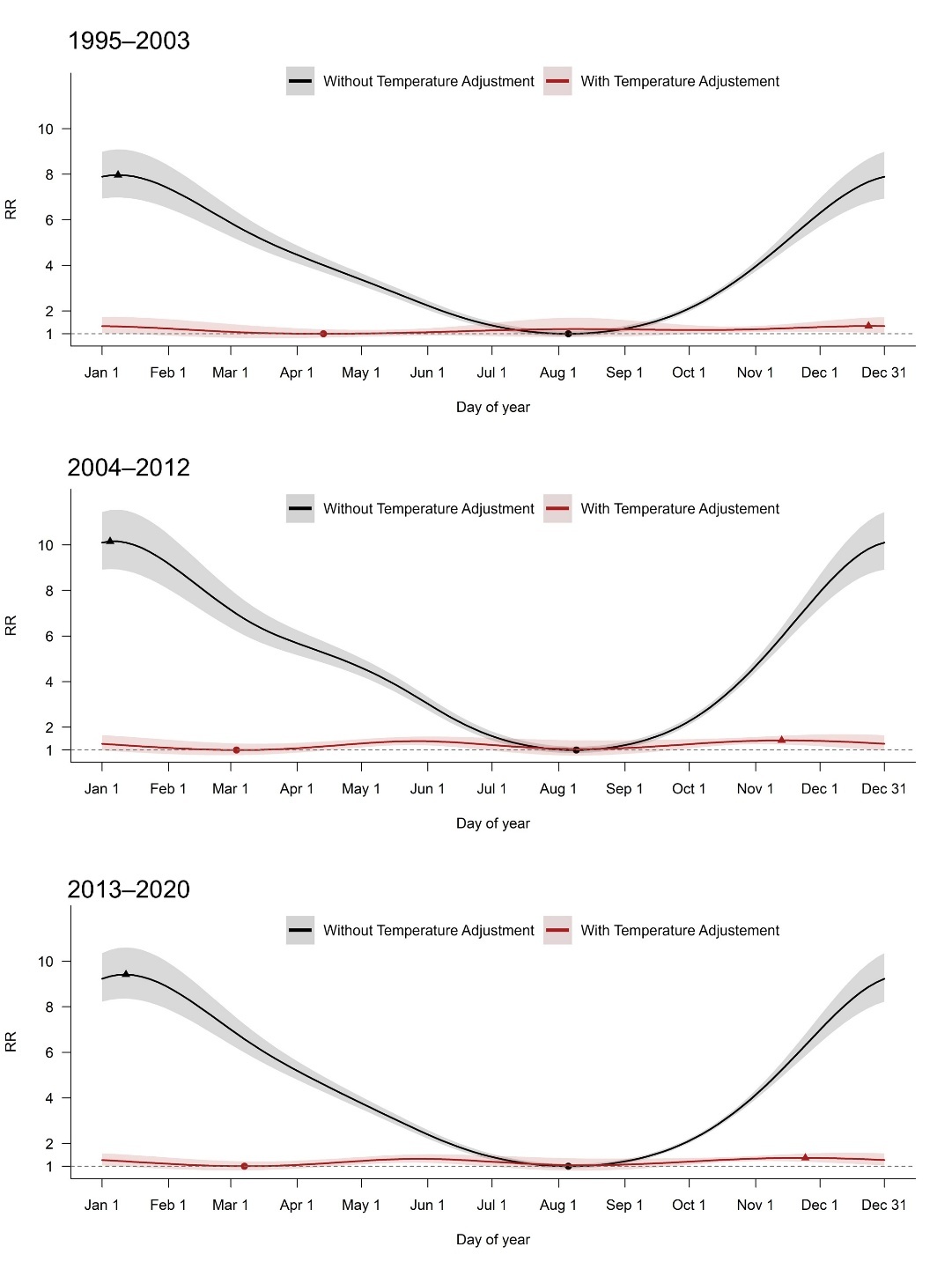


Seasonal patterns in bathtub-drowning mortality, modeled separately for 1995–2003, 2004–2012, and 2013–2020 using cyclic splines (4 degrees of freedom) for day of year, shown with and without adjustment for daily mean outdoor temperature. The temperature-adjusted curve includes a cross-basis function accounting for lagged outdoor temperature effects up to 21 days. Triangles indicate the seasonal peaks, and dots indicate the troughs.

**Fig. S4**. Relative risk of bathtub drowning deaths by outdoor temperature: comparison of lag 0 vs. lag 1 and lag 0 vs. lag 2


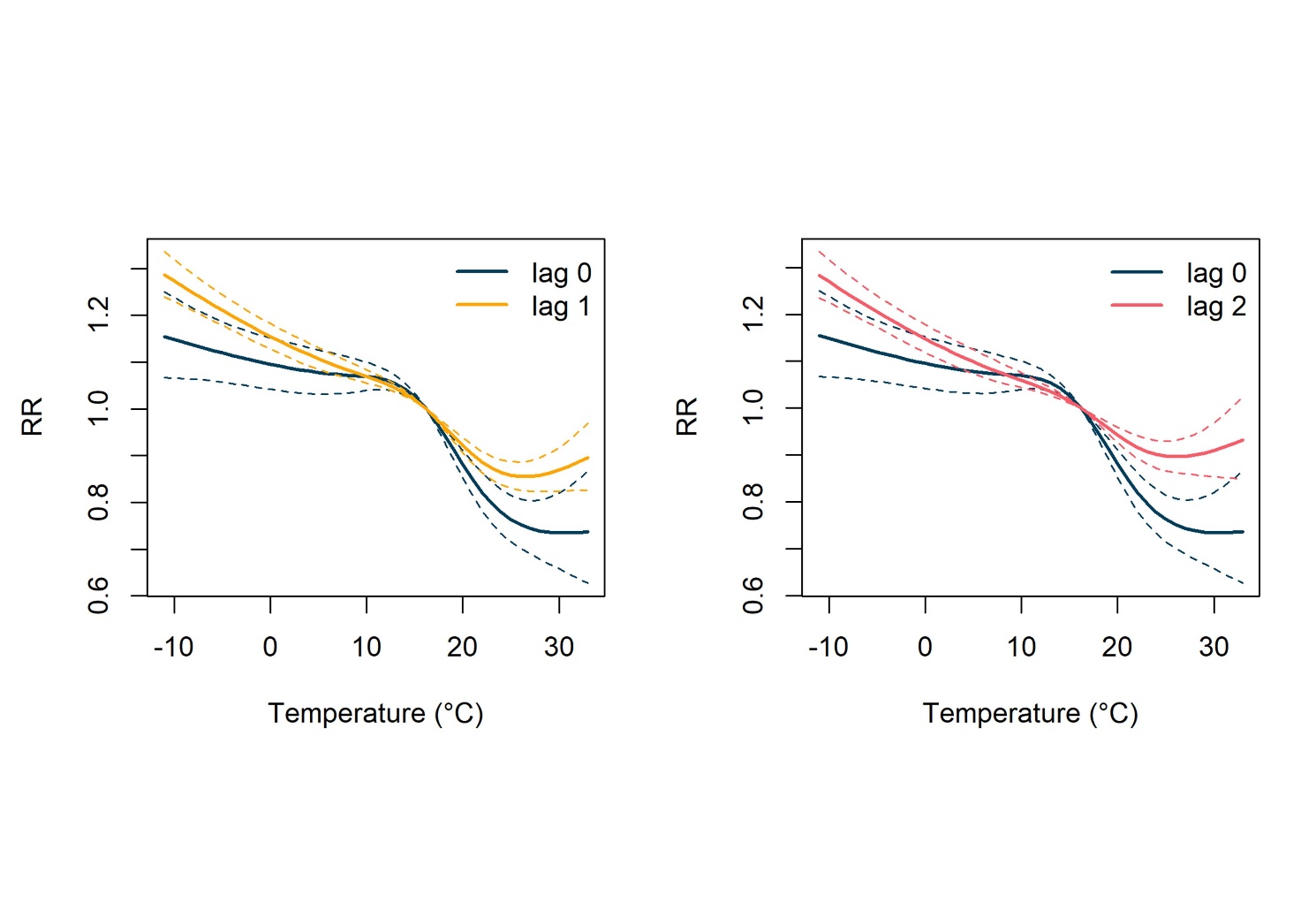


Dashed lines indicate 95% confidence intervals.

RR, relative risk.**Table S1**. Summary statistics of bathtub drowning mortality and daily mean outdoor temperature by prefecture (1995–2020)

| Prefecture | Annual Number of Deaths  (per million) | |  | Daily Mean Outdoor Temperature (°C) | |
| --- | --- | --- | --- | --- | --- |
|  | Median (IQR) | Range |  | Median (IQR) | Range |
| Hokkaido | 15.5 (11.8-24.3) | 7.5 to 41.5 |  | 9.8 (0.5-17.8) | -11.5 to 29.8 |
| Aomori | 15.5 (11.4-33.5) | 6.2 to 57.7 |  | 11.2 (2.4-18.5) | -7.8 to 30.1 |
| Iwate | 24.2 (21.8-32.2) | 15.6 to 37.3 |  | 10.8 (1.8-19) | -8.9 to 29.4 |
| Miyagi | 27.5 (24.7-31.1) | 15 to 35.3 |  | 13.4 (5.2-19.9) | -4.8 to 31.2 |
| Akita | 51.9 (39.9-67) | 29.8 to 94.4 |  | 12.3 (3.7-20.3) | -5.5 to 32.1 |
| Yamagata | 48.8 (36.9-59.3) | 26.5 to 71.6 |  | 12.4 (3.3-20.5) | -6.8 to 30.8 |
| Fukushima | 23.9 (16.3-37.3) | 11.4 to 47.5 |  | 13.8 (5.3-21) | -4.7 to 31.6 |
| Ibaraki | 23.3 (20.8-26.7) | 17.1 to 31.4 |  | 14.6 (6.6-21) | -2.8 to 31.3 |
| Tochigi | 12 (10.5-15.4) | 7.6 to 20.8 |  | 14.9 (6.4-21.6) | -2.8 to 31.7 |
| Gunma | 36 (26.7-54) | 12.4 to 64.3 |  | 15.4 (7.2-22.4) | -2.1 to 32.9 |
| Saitama | 9.1 (8-10.3) | 4.8 to 13.8 |  | 15.7 (7.5-22.4) | -2.3 to 32.7 |
| Chiba | 10.6 (9.6-12.4) | 7.8 to 19.5 |  | 16.8 (9.3-22.7) | 0.3 to 32.1 |
| Tokyo | 15.6 (13.9-17.5) | 11.4 to 19.7 |  | 17 (9.3-23.1) | 0 to 33.2 |
| Kanagawa | 64.3 (43.2-102.1) | 31.8 to 128.5 |  | 16.8 (9.4-22.6) | 0.3 to 32.2 |
| Niigata | 48.6 (46.1-52.8) | 40.8 to 61.4 |  | 14.3 (6.1-21.7) | -3.7 to 33 |
| Toyama | 60.3 (49.2-92.6) | 32.1 to 106.9 |  | 15 (6.6-22.1) | -3.5 to 33.1 |
| Ishikawa | 29.1 (23.2-39.2) | 14.4 to 60.6 |  | 15.5 (7.4-22.3) | -2.6 to 33.7 |
| Fukui | 53.2 (45-60) | 31.4 to 73.4 |  | 15.3 (6.8-22.6) | -1.9 to 32.4 |
| Yamanashi | 33.9 (26.6-61) | 17.9 to 82.6 |  | 15.6 (7.2-22.8) | -2.1 to 31.8 |
| Nagano | 40 (34.8-56.1) | 21.9 to 74.2 |  | 12.9 (3.4-20.8) | -6.8 to 30.9 |
| Gifu | 24.2 (20-45.7) | 16.1 to 76.5 |  | 16.7 (8.3-23.8) | -1.7 to 33.2 |
| Shizuoka | 31 (23-35.8) | 16.2 to 40.4 |  | 17.4 (10.4-23.3) | 1.2 to 31.9 |
| Aichi | 26 (23.3-33.4) | 19.1 to 47.2 |  | 16.7 (8.4-23.7) | -1.6 to 33.3 |
| Mie | 33.9 (29.1-40.1) | 14.5 to 58.7 |  | 16.6 (8.9-23.5) | -0.8 to 33.5 |
| Shiga | 26.8 (22.4-46.5) | 15.3 to 60.7 |  | 15.5 (7.3-22.8) | -1.9 to 31.3 |
| Kyoto | 5.7 (3.9-7.5) | 2.3 to 15.6 |  | 16.7 (8.3-23.8) | -1.2 to 32.6 |
| Osaka | 28.4 (24.4-36.4) | 19.9 to 42.2 |  | 17.5 (9.5-24.3) | -0.1 to 32.8 |
| Hyogo | 40.3 (31.6-47.4) | 21.5 to 53.3 |  | 17.7 (9.5-24.1) | -0.8 to 32.5 |
| Nara | 18.3 (15.9-22.7) | 5.8 to 30.6 |  | 15.6 (7.4-22.7) | -1.7 to 31.6 |
| Wakayama | 37.8 (32.8-42.5) | 22.3 to 52.6 |  | 17.5 (9.6-24) | -0.4 to 32.7 |
| Tottori | 32.3 (26.6-36.6) | 13.7 to 49.5 |  | 15.6 (7.7-22.5) | -3.5 to 32.3 |
| Shimane | 21.4 (17.2-36.8) | 6.5 to 58.7 |  | 15.5 (7.9-22.2) | -3.5 to 32.3 |
| Okayama | 28.8 (23.2-32.5) | 14.3 to 40.2 |  | 16.8 (8.4-23.9) | -2.2 to 32.3 |
| Hiroshima | 22.9 (19.2-33) | 13.9 to 42.5 |  | 17 (8.9-23.8) | -2.2 to 32.8 |
| Yamaguchi | 12.1 (10.4-14.7) | 4.9 to 32.8 |  | 16.3 (8.2-23.2) | -4.5 to 31.2 |
| Tokushima | 27 (12.5-41.9) | 7.3 to 64 |  | 17.4 (9.6-23.8) | -2.1 to 32.6 |
| Kagawa | 20.8 (17.6-23.6) | 9.9 to 41.6 |  | 17.2 (9.1-24) | -1.2 to 33 |
| Ehime | 24 (21.5-28.8) | 14.2 to 39 |  | 17.2 (9.6-23.7) | -0.7 to 31.9 |
| Kochi | 26.3 (21.7-36.3) | 14.8 to 49 |  | 18.1 (10.5-24.1) | -0.2 to 32.1 |
| Fukuoka | 59.3 (48.8-77.6) | 34.9 to 85.9 |  | 17.8 (10.4-24) | -2 to 32.8 |
| Saga | 31.5 (23.8-40.6) | 16.2 to 62.9 |  | 17.5 (9.7-24.1) | -3.3 to 32.5 |
| Nagasaki | 31.6 (18.3-56.2) | 13 to 70.1 |  | 18 (10.8-24) | -2.4 to 32.4 |
| Kumamoto | 18.6 (15.3-22) | 11.8 to 42.6 |  | 18 (10-24.5) | -3 to 31.9 |
| Oita | 22.5 (14.1-40.2) | 9 to 58.6 |  | 17.3 (9.8-23.4) | -1.7 to 31.7 |
| Miyazaki | 27.2 (13.6-43.3) | 6.8 to 62.7 |  | 18.5 (11.4-24.3) | 0.3 to 32 |
| Kagoshima | 18.7 (15.5-32.4) | 11.5 to 45.2 |  | 19.5 (12.5-25.3) | -0.4 to 31.7 |
| Okinawa | 1.9 (1.4-3.5) | 0 to 6.9 |  | 23.7 (19.7-27.8) | 9.2 to 31.4 |

IQR, interquartile range. The median and IQR for daily mean outdoor temperature were calculated based on data from the entire study period (1995–2020).

**Table S2**. Seasonality of bathtub drowning deaths with and without temperature adjustment by period

| Temperature  adjustment | Period | Impact | |
| --- | --- | --- | --- |
|  |  | Peak-to-trough ratio | Attributable fraction (%) |
|  |  | (95% CI) | (95% eCI) |
| Unadjusted | 1995–2003 | 7.97 (6.98, 9.09) | 75.5 (73.7, 77.1) |
|  | 2004–2012 | 10.16 (8.94, 11.55) | 79.6 (78.1, 80.9) |
|  | 2013–2020 | 9.41 (8.36, 10.60) | 77.5 (76.2, 78.7) |
|  |  |  |  |
| Adjusted | 1995–2003 | 1.34 (1.09, 1.74) | 16.2 (12.0, 24.3) |
|  | 2004–2012 | 1.42 (1.26, 1.69) | 18.8 (14.3, 33.2) |
|  | 2013–2020 | 1.37 (1.23, 1.60) | 17.1 (13.5, 30.0) |

CI, confidence interval; eCI, empirical confidence interval.**Table S3**. Projected population and proportion of individuals aged ≥65 years across three scenarios

| Decade | High Population Projection | |  | Intermediate Population Projection | |  | Low Population Projection | |
| --- | --- | --- | --- | --- | --- | --- | --- | --- |
|  | Population (million) | Population  aged ≥65 years (%) |  | Population (million) | Population  aged ≥65 years (%) |  | Population (million) | Population  aged ≥65 years (%) |
| 2020s | 124.4 | 29.7 |  | 123.5 | 29.5 |  | 122.6 | 29.4 |
| 2030s | 119.8 | 32.4 |  | 116.9 | 32.3 |  | 114.2 | 32.1 |
| 2040s | 113.9 | 35.8 |  | 109.2 | 36.0 |  | 104.9 | 36.2 |
| 2050s | 107.5 | 36.7 |  | 100.9 | 37.5 |  | 95.0 | 38.2 |
| 2060s | 101.1 | 36.6 |  | 92.0 | 38.3 |  | 84.4 | 39.7 |

Population and the proportion of individuals aged ≥65 years were presented as mean values for each decade.

Population scenarios were classified as high, intermediate, or low based on assumptions of birth and mortality rates: high birth rate and low mortality (high population), medium birth and mortality rates (intermediate population), and low birth rate with high mortality (low population).
